# Building robust, proportionate, and timely approaches to regulation and evaluation of digital mental health technologies

**DOI:** 10.1016/S2589-7500(24)00215-2

**Published:** 2024-11-15

**Authors:** Gareth Hopkin, Richard Branson, Paul Campbell, Holly Coole, Sophie Cooper, Francesca Edelmann, Grace Gatera, Jamie Morgan, Mark Salmon

**Affiliations:** National Institute for Health and Care Excellence, Manchester, UK; Medicines and Healthcare products Regulatory Agency, London, UK; National Institute for Health and Care Excellence, Manchester, UK; Medicines and Healthcare products Regulatory Agency, London, UK; Wellcome Trust, London, UK; National Institute for Health and Care Excellence, Manchester, UK

## Abstract

Demand for mental health services exceeds available resources globally, and access to diagnosis and evidence-based treatment is affected by long delays. Digital mental health technologies present an opportunity to reimagine the delivery of mental health support by providing innovative, effective, and tailored approaches that meet people’s individual preferences and goals. These technologies also present new challenges, however, and efforts must be made to ensure they are safe and effective. The UK Medicines and Healthcare products Regulatory Agency and the National Institute for Health and Care Excellence have launched a partnership, funded by Wellcome, that explores regulation and evaluation of digital mental health technologies. This Viewpoint describes a series of key challenges across the regulatory and health technology assessment pathways and aims to facilitate discussions to ensure that approaches to regulation and evaluation are informed by patients, the public, and professionals working within mental health. We invite partners from across the mental health community to engage with, collaborate with, and provide scrutiny of this project to ensure it delivers the best possible outcomes.

## Introduction

Digital mental health technologies (DMHTs) are becoming well established within mental health services and through direct-to-consumer models.^1,2^ Apps and websites that target common mental ill health such as anxiety and depression, have expanded most rapidly;^3^ however, use of DMHTs is also growing in relation to other conditions and functions (eg, triage, diagnosis, treatment, and monitoring), with some DMHTs using more complex technology (eg, generative artificial intelligence [AI]).^4^

DMHTs present opportunities to improve access to and quality of mental health care.^5^ Due to their scalable nature, DMHTs can support health services to bridge the gap between the demand for interventions and the available workforce. These technologies can also enable earlier identification of mental ill health, improvements to existing treatment, and better access to services.

Many challenges and risks are associated with DMHTs, however.^6^ In an evolving market, who technologies are aimed at, how they function, and whether claims of safety and effectiveness are evidence-based are not clear. Health and social care professionals, patients, and the public also have concerns about user engagement and experience and the impact that negative experiences might have on future help-seeking behaviour.

## Ensuring effective regulation and evaluation

In the UK, the Medicines and Healthcare products Regulatory Agency (MHRA) is responsible for regulating medical devices, including DMHTs that are considered software (and AI) as a medical device (SaMD). The MHRA is developing the future UK medical device regulatory framework^7^ and working on a SaMD Change Programme.^8,9^ These initiatives will provide new UK legal text and guidance documents that outline requirements for developing SaMD.

Some DMHTs are designed to be used within the National Health Service (NHS), and these will be assessed by the National Institute for Health and Care Excellence (NICE), England’s health technology assessment (HTA) agency. NICE is expanding its assessment of digital health technologies through the Early Value Assessment programme to ensure that patients benefit from promising technologies as early as possible.^10^

A whole-system approach is needed to ensure safe and effective use of DMHTs, as the remit of the MHRA and NICE has boundaries that reduce the capability to oversee all potential concerns or risks associated with DMHT use. For example, the MHRA only regulate SaMD, but many DMHTs do not qualify as medical devices and are available to consumers. Furthermore, technologies will only be eligible for NICE assessment if they are suitably regulated by the MHRA. This approach will require collaborative engagement across public agencies to ensure that services that DMHTs are integrated into provide high-quality care and that health-care commissioners and professionals can select appropriate digital tools. Furthermore, other organisations that operate within the DMHT ecosystem might have a role in supporting developers to bring products to market or to provide advice on the most appropriate technologies for users and health services.

## MHRA and NICE partnership

Due to the distinct nature of mental health and digital technologies, stakeholders have called for clarity on how regulatory requirements apply to DMHTs.^11,12^ Wellcome has funded a 3-year project, spearheaded by the MHRA and in partnership with NICE, to respond to these calls.^13^ This innovative approach to funding allows the MHRA and NICE to work closely together and engage with a range of stakeholders in this high-priority clinical speciality. The partnership launched in May, 2023, and will publish findings and guidance over the life of the project until it concludes in early 2026. The key stages across the regulatory and HTA pathway that will be explored in the context of DMHTs during the project and where further guidance might be needed are outlined in the figure.

First, developers must provide a clear account of a DMHT’s intended purpose. MHRA guidance on crafting an intended purpose statement for SaMD recommends including how the DMHT functions, what it aims to achieve, who will operate the software and in what environment, and who will benefit.^14^ To provide this detail within an intended purpose, developers will need to engage early with adopters and end users to ensure that they are able to integrate their technologies into existing systems and to understand the pathways in which their technologies will be implemented. Early engagement might also include understanding whether pathways will need to be modified to accommodate adoption. In the authors’ experience, intended purpose statements for many DMHTs are inadequate. Intended purpose statements can also become inaccurate when a developer expands the purpose of their technology without updating key documentation. In some cases, this change in purpose can have a substantial effect on users and the risks of using the technology.

Second, developers are responsible for establishing whether their DMHT qualifies as SaMD and its appropriate classification. MHRA guidance on qualification and classification of SaMD is available,^15^ but it does not include examples for mental health. These judgements can be challenging because DMHTs might target symptoms that are linked to mental health (eg, sleep, mood, and stress) but are not themselves medical conditions. Similarly, whether psychoeducation or other therapeutic behavioural interventions supported by digital versions of offline manuals or workbooks with little personalisation and interactivity should qualify as SaMD is ambiguous. These decisions will also have implications for the wider system as a group of DMHTs could play a role within health services but not qualify as SaMD. NICE and other partners will need to consider whether these DMHTs should be evaluated and recommended for use and how their risks can be managed during deployment.

Third, developers must ensure DMHTs have adequate clinical and economic evidence to show that the benefits outweigh the risks.^16^ NICE will publish recommendations on using certain DMHTs within the NHS based on their effectiveness and cost-effectiveness compared with established care, but DMHTs might not have sufficient clinical or economic evidence to meet regulatory requirements or for NICE to recommend them for routine use.^17^ The NICE Evidence Standards Framework provides some information on what good evidence for digital health technologies look like;^17^ however, more clarity is needed as to how DMHTs should be classified within this framework, and more detail could be provided on specific aspects of high-quality study design (eg, on the appropriate quasiexperimental method and the most relevant patient-reported outcome measures and comparators). An opportunity also exists to consider whether innovative approaches to developing evidence could reduce uncertainty in decision making. For example, platform trials, because of their adaptive design, allow evaluation of DMHTs over time, and can compare them with multiple comparator interventions simultaneously.^18^

Fourth, developers must ensure that DMHTs remain safe and effective after initial assessment, through robust post-market surveillance and lifecycle assessment. In 2023, the MHRA published guidance on reporting adverse incidents involving SaMD through vigilance systems, including references to direct and indirect harms from using DMHTs.^19^ New approaches within NICE that recommend evidence generation while DMHTs are used in real-world settings could complement these feedback mechanisms. How to monitor possible harms and whether new data sources can be harnessed to strengthen existing reporting systems (eg, Yellow Card reporting) and share information more effectively need to be considered. This assessment could include use of routinely collected data after adoption to support real-world surveillance and novel approaches to monitor product reviews in app stores and other forums. These approaches could have benefits but would also present practical challenges (eg, ensuring that the use of DMHTs is adequately coded within patient records and that data collected during interactions with DMHTs can be integrated into outcome sets).

Fifth, the number of DMHTs available to the public, patients, and health-care professionals is large and likely to increase.^4^ This project will provide tailored guidance to help each of these groups understand the aforementioned regulatory and evaluation challenges and support them to choose safe and effective products suitable for their needs.

Finally, there is a global market for DMHTs, and many regulatory and HTA agencies are facing similar challenges. Opportunities are available to move towards international alignment where possible and to provide clarity on where different approaches are needed. For example, regulators might also take differing approaches to assessing whether evidence can be generalised across similar types of DMHTs with some agencies, such as the US Food and Drug Administration, having separate pathways for products that do meet criteria for substantial equivalence to a previously approved product.^20^ These differences could have implications for developers when planning evidence generation to support market access in different jurisdictions. The MHRA is a member of the International Medical Device Regulators Forum, which aims to enhance regulatory harmonisation and convergence. Through the project and other research and collaboration initiatives, the MHRA and NICE have engaged with international regulators and HTA agencies and will continue to do so to share learnings and explore further international harmonisation. The project also aims to explore how countries with less established regulatory pathways can develop frameworks to allow safe access to DMHTs.

## Incorporating the perspectives of people with lived experience

In recent decades, calls have been made for the public and patients to have greater involvement in decision-making processes about their health care than they have had in the past.^21,22^ The MHRA and NICE recognise the need for meaningful involvement of the public and patients in their work and have ensured that the perspectives of people with lived experience are represented across the project.^23,24^ The MHRA has also completed research with the wider public to explore perceptions and attitudes towards DMHTs, their benefits and risks, and the role of regulation and evaluation.^25^

## Reflections from lived experience advisers on the project board

Collaborative research is key to Wellcome’s aim to create a world where nobody is held back by mental ill health. This project has collaborated with and involved people with lived experience to ensure the outcomes are relevant and, ultimately, to help get the right treatments to those who need them.

Lived experience advisers have been recruited to the project board, which comprises representatives from the MHRA, NICE, and Wellcome. Within these meetings, advisers can influence the project’s strategic direction. Involvement is not about one form of expertise taking over from another; it is about people with differing expertise collaborating and complementing each other.

Before the first board meeting in September, 2023, advisers had a comprehensive induction, including meeting individually with members of the project team and other lived experience advisers to review project details and ask questions. Having a defined point of contact during involvement activities is important to provide familiarity, ensure lived experience advisers are comfortable throughout the project, and foster collaboration, which the process achieved according to the lived experience advisers. The introductory meetings also allowed lived experience advisers working across different parts of the project to meet.

During these introductory meetings, flexible involvement was a central theme, which is important to ensure that the project environment is supportive of the individual needs and preferences of lived experience advisers.^26^ Here, advisers have been involved through one-to-one meetings, a larger group discussion, emails, and document review. Each meeting has been characterised by inclusive and accessible engagement.

## Conclusion

For the potential of DMHTs to be realised, stakeholders within local, national, and international mental health communities must work together. Industry has a key role in developing technologies that target unmet needs and maximise benefits. Regulators and HTA agencies need to clarify what is expected of these developers to ensure that products are safe and effective and can be used for the greatest benefit. Crucially, all DMHT development processes must be codesigned with people with lived experience, their support networks, and professionals who deliver care.

The MHRA and NICE, supported by Wellcome, invite relevant stakeholders to engage, collaborate, and provide invaluable scrutiny to ensure our project contributes to delivering the best possible outcomes for people facing challenges with mental health within sustainable new models of care. Together, this collaboration will help achieve the ambition of bringing a wave of change in mental health care with the support of DMHTs in an environment where regulation facilitates the choice of safe and effective tools.

Through the project, the MHRA and NICE will continue to organise engagement activities to ensure that the voices of a diverse group of stakeholders can be heard and can inform the project and influence outputs. These activities include a series of roundtables and other targeted events that aim to bring together stakeholders and seek consensus on future directions for regulation and evaluation. As outputs from the project are published, the project team will also develop learning programmes and other educational materials specific to DMHT regulation and evaluation according to the needs of people from different professional groups, people with lived experience, and the public.

## Figures and Tables

**Figure:**
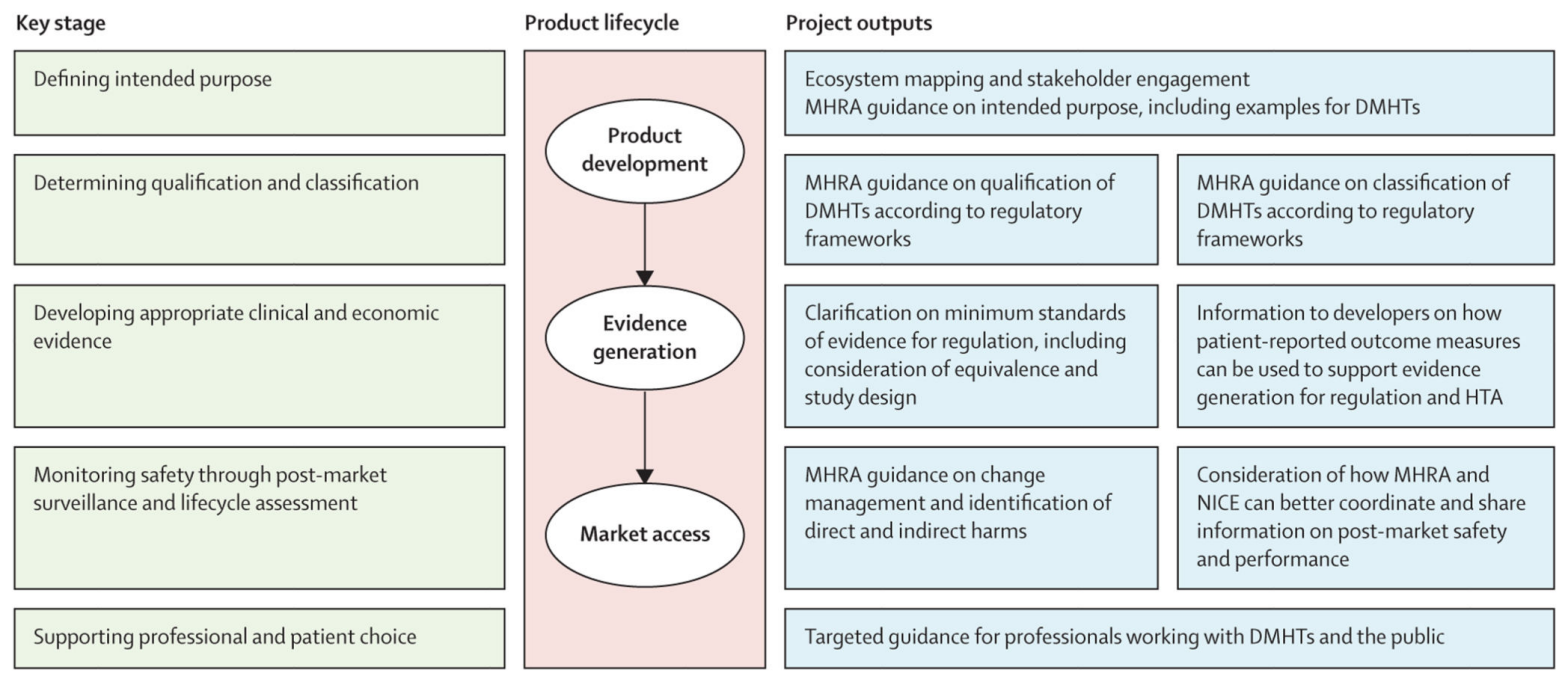
Key stages across the regulatory and HTA pathway and project outputs. DMHTs=digital mental health technologies. HTA=health technology assessment. MHRA=Medicines and Healthcare products Regulatory Agency. NICE=National Institute for Health and Care Excellence.
